# Reversal of diabetic-induced myopathy by swimming exercise in pregnant rats: a translational intervention study

**DOI:** 10.1038/s41598-022-10801-z

**Published:** 2022-05-05

**Authors:** Bruna B. Catinelli, Patrícia S. Rossignoli, Juliana F. Floriano, Aline M. Carr, Rafael G. de Oliveira, Nilton J. dos Santos, Lara C. C. Úbeda, Maria Angélica Spadella, Raghavendra L. S. Hallur, Luis Sobrevia, Sérgio L. Felisbino, Iracema M. P. Calderon, Angélica M. P. Barbosa, Marilza V. C. Rudge, The Diamater Study Group, M. V. C. Rudge, A. M. P. Barbosa, I. M. P. Calderon, L. Sobrevia, F. P. Souza, B. Berghmans, L. Thabane, B. Junginger, C. F. O. Graeff, C. G. Magalhães, R. A. Costa, S. A. M. Lima, M. R. Kron-Rodrigues, S. L. Felisbino, W. Barbosa, F. J. Campos, G. Bossolan, J. E. Corrente, H. R. C. Nunes, J. Abbade, P. S. Rossignoli, C. R. Pedroni, A. N. Atallah, Z. I. K. J. Di Bella, S. M. M. Uchoa, M. A. H. Duarte, E. A. Mareco, M. E. Sakalem, N. Martinho, L. S. R. Hallur, D. R. A. Reyes, F. C. B. Alves, J. P. C. Marcondes, C. B. Prudencio, F. A. Pinheiro, C. I. SartorãoFilho, S. B. C. V. Quiroz, T. Pascon, S. K. Nunes, B. B. Catinelli, F. V. D. S. Reis, R. G. Oliveira, S. Barneze, E. M. A. Enriquez, L. Takano, A. M. Carr, A. B. M. Magyori, L. F. Iamundo, C. N. F. Carvalho, M. Jacomin, R. E. Avramidis, A. J. B. Silva, M. I. G. Orlandi, T. D. Dangió, H. C. M. Bassin, M. L. S. Takemoto, T. D. Caldeirão, N. J. Santos, I. O. Lourenço, J. Marostica de Sá, I. P. Caruso, L. T. Rasmussen, G. A. Garcia, G. T. A. Nava, C. P. Marques, D. G. Bussaneli, V. K. C. Nogueira, C. V. C. Rudge, F. Piculo, G. M. Prata, V. P. Barbosa

**Affiliations:** 1 Postgraduate Program on Tocogynecology, Department of Gynecology and Obstetrics, Botucatu Medical School, São Paulo State University (UNESP), Botucatu, São Paulo Brazil; 2 Department of Physiotherapy and Occupational Therapy, School of Philosophy and Sciences, São Paulo State University (UNESP), Marília, São Paulo Brazil; 3 Laboratory of Extracellular Matrix Biology, Department of Structural and Functional Biology, Institute of Biosciences of Botucatu, São Paulo State University (UNESP), Botucatu, São Paulo Brazil; 4 Department of Structural and Functional Biology, Institute of Biology (IB), UNICAMP, University of Campinas (UNICAMP), Campinas, São Paulo Brazil; 5 University of Marília (UNIMAR), Marília, São Paulo Brazil; 6 Human Embryology Laboratory, Marília Medical School (FAMEMA), Marília, São Paulo Brazil; 7 Pravara Institute of Medical Sciences (Deemed to be University), Loni, Rahata Taluk, Ahmednagar District, Maharashtra 413736 India; 8 Cellular and Molecular Physiology Laboratory (CMPL), Department of Obstetrics, Division of Obstetrics and Gynaecology, School of Medicine, Faculty of Medicine, Pontificia Universidad Católica de Chile, 8330024 Santiago, Chile; 9 Department of Physiology, Faculty of Pharmacy, Universidad de Sevilla, 41012 Seville, Spain; 10 University of Queensland Centre for Clinical Research (UQCCR), Faculty of Medicine and Biomedical Sciences, University of Queensland, Herston, QLD 4029 Australia; 11 Division of Pathology, Department of Pathology and Medical Biology, University of Groningen, University Medical Center Groningen (UMCG), 9713GZ Groningen, The Netherlands; 12 Department of Physics, Institute of Biosciences, Letters and Exact Sciences (IBILCE), Multiuser Center for Biomolecular Innovation (CMIB), São Paulo State University “Júlio de Mesquita Filho” (UNESP), São José do Rio Preto, São Paulo 15054-000 Brazil; 13 Pelvic Care Center Maastricht, Maastricht University Medical Center, Maastricht, The Netherlands; 14 Department of Clinical Epidemiology and Biostatistics, McMaster University, Hamilton, ON Canada; 15 Department of Gynecology, Pelvic Floor Center Charité, Charité University Hospital, Berlin, Germany; 16 POSMAT-Post-Graduate Program in Materials Science and Technology, School of Sciences, São Paulo State University (UNESP), Bauru, São Paulo Brazil; 17 Medical School of Botucatu, São Paulo State University (UNESP), Botucatu, São Paulo Brazil; 18 Department of Nursing, Botucatu Medical School (FMB), São Paulo State University (UNESP), Botucatu, São Paulo Brazil; 19 Stricto Sensu Graduate Program in Nursing at UNIVERITAS, Guarulhos University (UNG), Guarulhos, São Paulo Brazil; 20 Department of Clinical Medicine, Botucatu Medical School, São Paulo State University (UNESP), Av. Prof. Mário Rubens Guimarães Montenegro, s/n-UNESP, Botucatu, São Paulo Brazil; 21 Department of Biostatistics, Botucatu Institute of Biosciences, São Paulo State University/UNESP, São Paulo, tistics, Botucatu Institute of Biosciences Brazil; 22 Discipline of Evidence-Based Medicine, Paulista School of Medicine-Federal University of São Paulo (EPM-UNIFESP), São Paulo, Brazil; 23 Department of Gynecology, Federal University of São Paulo, UNIFESP, São Paulo, Brazil; 24 Department of Physical Therapy, Universidade Católica de Pernambuco (UNICAP), Recife, PE Brazil; 25 Department of Operative Dentistry, Endodontics and Dental Materials, Bauru School of Dentistry, University of São Paulo, São Paulo, Brazil; 26 University of Western São Paulo (UNOESTE), Presidente Prudente, São Paulo Brazil; 27 Department of Anatomy, CCB, State University of Londrina (UEL), Campus Universitário s/n , Caixa Postal 10011, Londrina, Parana 86057-970 Brazil; 28 University Center of Associated Colleges, São João da Boa Vista, São Paulo Brazil; 29 Regional University Center of Espírito Santo do Pinhal, UNIPINHAL, Espírito Santo do Pinhal, São Paulo Brazil; 30 Universidade do Sagrado Coração (USC), Bauru, São Paulo Brazil; 31 Department of Physical Education, Institute of Biosciences of Rio Claro, São Paulo State University (UNESP), Rio Claro, São Paulo Brazil; 32 Independent Researcher, Assis, São Paulo Brazil; 33 Department of Morphology and Pediatric Dentistry, Araraquara School of Dentistry, São Paulo State University (UNESP), Rua Humaitá, 1680, Araraquara, São Paulo 14801-903 Brazil; 34 Independent Researcher, Botucatu, SP Brazil; 35 Health Sciences Center, University of the Sacred Heart (USC), Bauru, São Paulo Brazil; 36 Universidade de Marília (UNIMAR), Marília, São Paulo Brazil

**Keywords:** Physiology, Muscle, Gestational diabetes

## Abstract

Gestational diabetes mellitus (GDM) plus rectus abdominis muscle (RAM) myopathy predicts long-term urinary incontinence (UI). Atrophic and stiff RAM are characteristics of diabetes-induced myopathy (DiM) in pregnant rats. This study aimed to determine whether swimming exercise (SE) has a therapeutic effect in mild hyperglycemic pregnant rats model. We hypothesized that SE training might help to reverse RAM DiM. Mild hyperglycemic pregnant rats model was obtained by a unique subcutaneous injection of 100 mg/kg streptozotocin (diabetic group) or citrate buffer (non-diabetic group) on the first day of life in Wistar female newborns. At 90 days of life, the rats are mated and randomly allocated to remain sedentary or subjected to a SE protocol. The SE protocol started at gestational day 0 and consisted of 60 min/day for 6 days/week in a period of 20 days in a swim tunnel. On day 21, rats were sacrificed, and RAM was collected and studied by picrosirius red, immunohistochemistry, and transmission electron microscopy. The SE protocol increased the fiber area and diameter, and the slow-twitch and fast-twitch fiber area and diameter in the diabetic exercised group, a finding was also seen in control sedentary animals. There was a decreased type I collagen but not type III collagen area and showed a similar type I/type III ratio compared with the control sedentary group. In conclusion, SE during pregnancy reversed the RAM DiM in pregnant rats. These findings may be a potential protocol to consider in patients with RAM damage caused by GDM.

## Introduction

Gestational diabetes mellitus (GDM) is known as a serious global health problem^1^. GDM can cause damage to the skeletal muscle and extracellular matrix (ECM) health and function, i.e. maternal hyperglycemic myopathy^2–4^. Maternal GDM leads to long-term pelvic floor muscle (PFM) dysfunction and urinary incontinence (UI)^5–7^, having pregnancy specific-urinary incontinence as a risk factor^5^. It is unknown how this link occurs^4^ and how swimming exercise (SE) may alleviate this damage in mild hyperglycemic pregnancy (MHP).

Rat models of MHP also revealed myopathy of the PFM and rectus abdominis muscle (RAM) induced by diabetes (DiM). DiM characterized by muscle atrophy, a shift in the maternal fiber type composition, increased collagen deposition, and higher collagen type I/III ratio ^8–11^. The latter characteristics mimic those found in human hyperglycemic-associated PFM and RAM myopathy and are considered a profile of skeletal muscle injury caused by GDM during pregnancy in humans ^11–13^. Despite these observations, the link between GDM and maternal hyperglycemic myopathy remains largely unexplored and without an effective treatment ^14^.

Exercise has beneficial impacts on maternal glycaemia control and several lifestyle interventions have clarified mechanisms underlying GDM for preventing or minimizing this disease’s associated complications ^8,9,14–18^. However, different exercise patterns improve maternal glucose control in some but not all pregnant women with GDM ^19–21^. Thus, lifestyle interventions during pregnancy alone may not be sufficient to decrease the risk of developing GDM-associated alterations in maternal health ^22–27^.

Considering that pregnant women should avoid high-impact exercises with the risk of falling and with the risk of abdominal trauma, SE is considered ideal for pregnant women. Also, SE in a low-moderate intensity is effective in promoting glycemic control and preventing GDM ^21,24,27^. In addition, SE and resistance training influence the gastrocnemius and soleus muscles myopathy in rat models of type 2 diabetes mellitus (T2DM)^28,29^. However, there is limited evidence describing the effect of SE on diabetic myopathy in GDM ^22,30^. There are no studies revealing whether the exercise intervention during pregnancy recovers the muscle damage associated with GDM, even considering the regenerative skeletal muscle fibers potential ^31^.

The aim of this study was to determine whether SE has a therapeutic effect in MHP rats model resulting in attenuation of RAM DiM. We hypothesized that SE training would mitigate RAM DiM, thus reversing the RAM injury. A potential reversal of RAM DiM by SE may improve the handling of maternal hyperglycemic myopathy in women that developed GDM.

## Results

**Figure 1:**
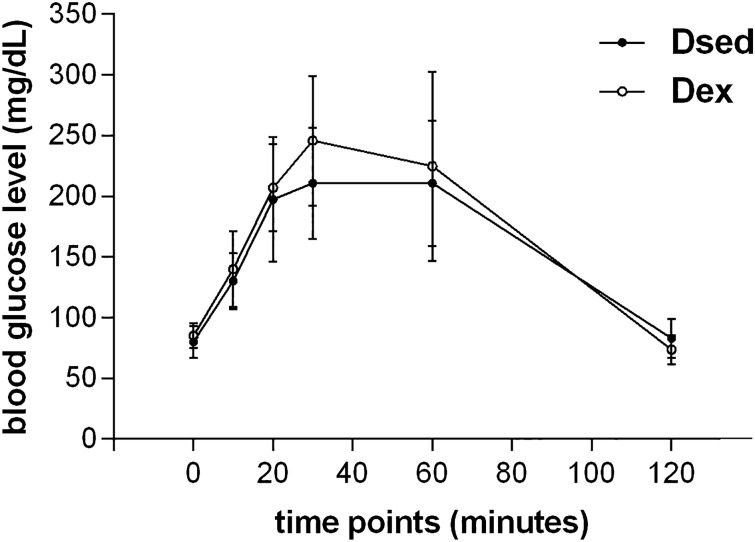
Oral glucose tolerance test. Blood glucose level before the test and 10, 20, 30, 60 and 120 min after the administration of intragastric glucose solution in Dsed and Dex groups.

The experimental design included three control groups, i.e., non-diabetic sedentary (NDsed), non-diabetic exercised (NDex), and diabetic sedentary allowing the analysis of the SE intervention in DiM in the authentic study group: the diabetic exercised (Dex). The OGTT showed that Dsed and Dex groups had two or more blood glucose measurements > 140 mg/dl. These data confirm that the generation of MHP rats model was efficient, including the animals in the experimental groups. The aquatic exercise practice during pregnancy did not promote changes in blood glucose levels (Fig. 1).

**Figure 2:**
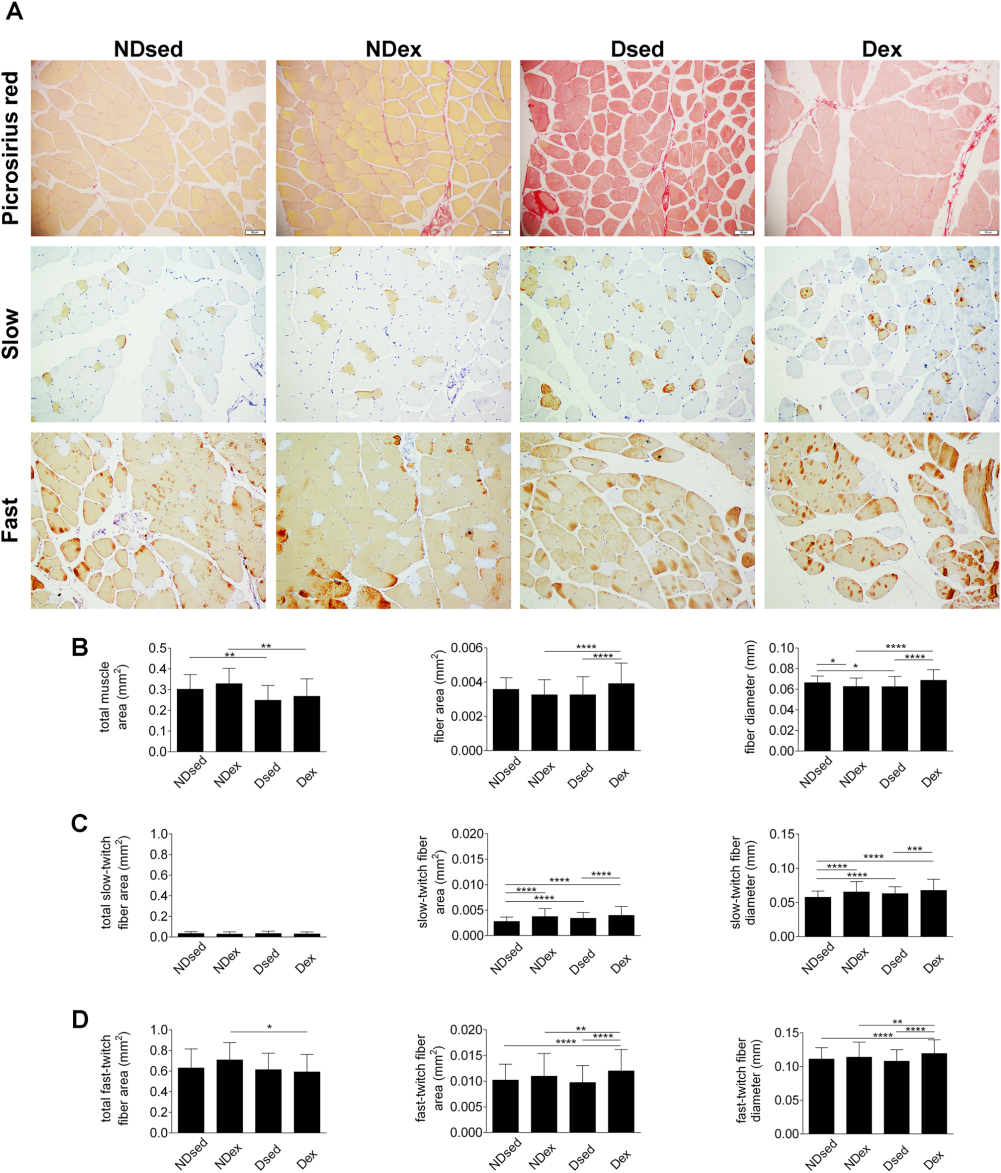
Histological sections of rat rectus abdominis muscle (RAM), obtained from CellSens Dimension (Olympus Corporation®) Version 1.16 image analysis software—(https://www.olympus-lifescience.com/en/software/cellsens/). **(A**) RAM samples were taken from non-diabetic sedentary (NDsed), non-diabetic exercised (NDex), diabetic sedentary (Dsed) and diabetic exercised (Dex) rats as described in “Materials and methods”. RAM samples were stained with picrosirius red and immunohistochemistry against myosin heavy chain of slow and fast RAM fibers. **(B)** Picrosirius red staining for total muscle area, fiber area, and fiber diameter in RAM samples as in (**A**). (**C**) Immunohistochemistry for total slow-twitch fiber area,slow-twitch fiber area, and slow-twitch fiber diameter as in (**A**). (**D**) Immunohistochemistry for total fast-twitch fiber area,fast-twitch fiber area, and fast-twitch fiber diameter as in (**A**). Values are means ± S.D. (n = 5 animals/group. *p < 0.05, **p < 0.01, ***p < 0.001 and ****p < 0.0001. Scale bar: 50 µm. Magnification: ×20.

The RAM DiM was confirmed in diabetic sedentary rats, demonstrated by the lower total muscle area and the fiber diameter compared with NDsed group (Fig. 2A,B). However, the fiber area was unaltered in Dsed compared with NDsed group.

**Figure 3:**
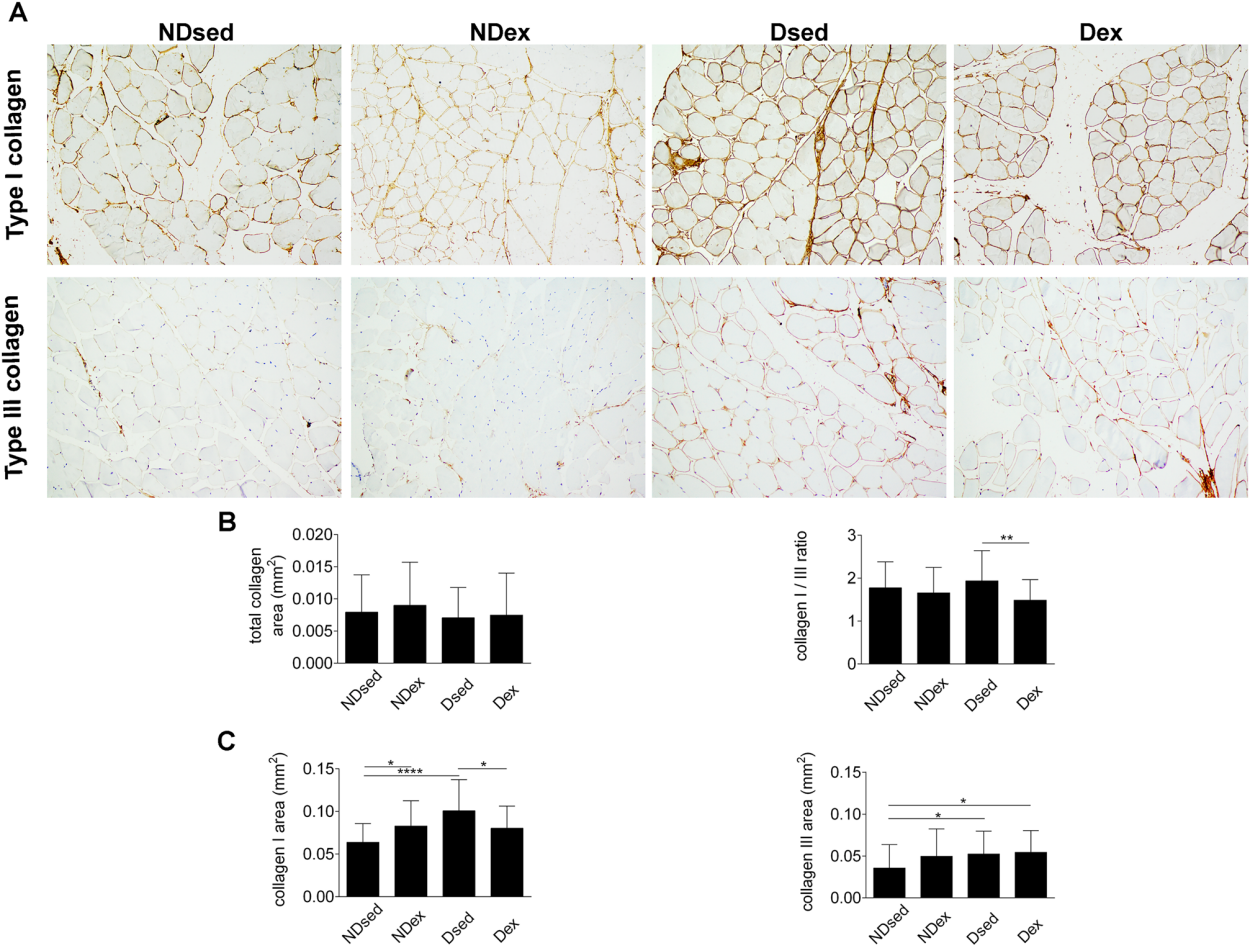
Immunohistochemical sections of rat rectus abdominis muscle (RAM), obtained from CellSens Dimension (Olympus Corporation^®^) Version 1.16 image analysis software—(https://www.olympus-lifescience.com/en/software/cellsens/). (**A**) RAM samples were taken from non-diabetic sedentary (NDsed), non-diabetic exercised (NDex), diabetic sedentary (Dsed) and diabetic exercised (Dex) rats as described in Materials and methods. RAM samples were stained with Picrosirius red and immunohistochemistry using antibodies against type I and III collagen on RAM fibers. **(B)** Picrosirius red staining for total collagen area and immunohistochemistry for type I/III ratio. (**C**) Immunohistochemistry for type I and III collagen area as in (**A**). Values are means ± S.D. (n = 5 animals/group. *p < 0.05, **p < 0.01, ***p < 0.001 and ****p < 0.0001. Scale bar: 50 µm. Magnification: ×20.

The immunohistochemical analysis showed that the slow-twitch fiber area and diameter (Fig. 2C) were higher in Dsed compared with NDsed group. However, the fast-twitch fiber area and diameter (Fig. 2D) were unaltered in Dsed compared with NDsed group. The total collagen area and collagen I/III ratio (Fig. 3B) were unaltered in Dsed compared with NDsed group. However, type I collagen area and type III collagen area (Fig. 3C) were higher in Dsed compared with NDsed group.

**Figure 4:**
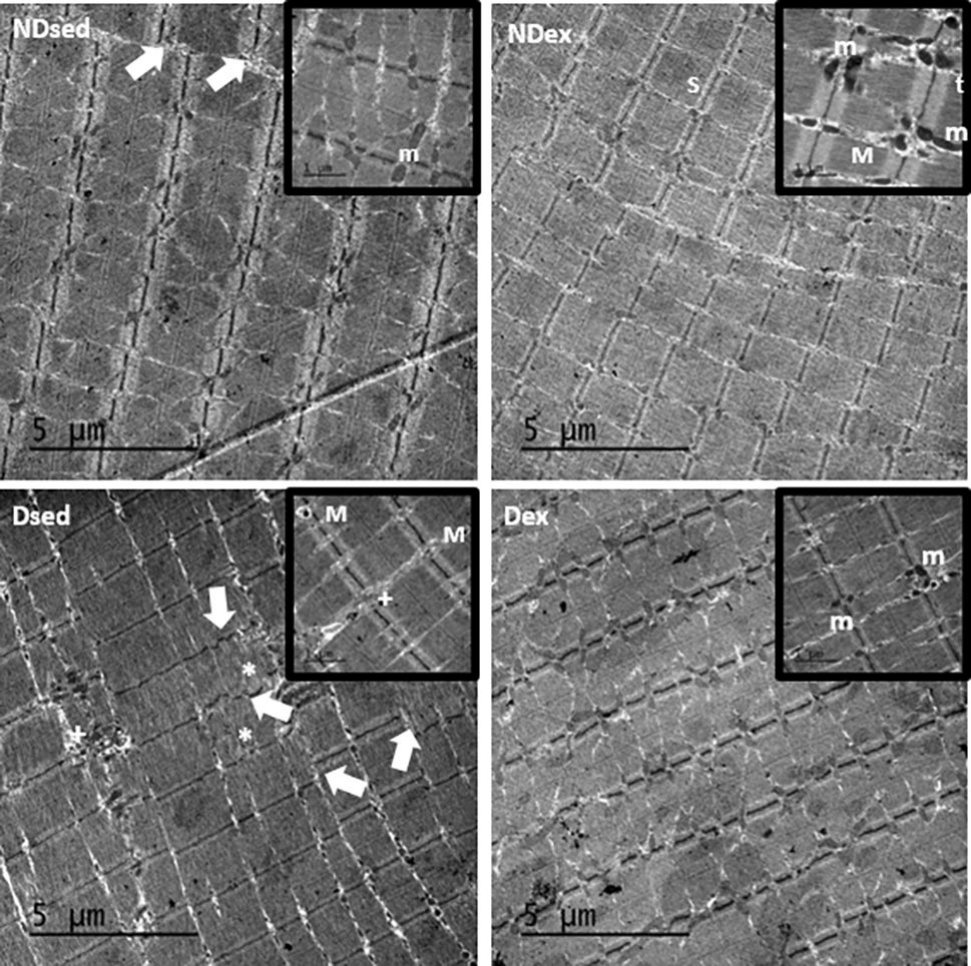
Electron micrographs of rectus abdominis muscle (RAM). Samples of RAM were obtained from non-diabetic sedentary (NDsed), non-diabetic exercised (NDex), diabetic sedentary (Dsed), and diabetic exercised (Dex) rats. Magnifications (20.000 x) show a detailled area of the micrograph. The micrographs show disorganized Z lines (white arrows), sarcomeres disruption areas (asterisk), intermyofibrillar mitochondria (m), myelin figures (M), organized triads (t) and an increase in collagen deposition ( +). Scale bar: 5 µm.

Electron micrographs of RAM from Dsed group showed disorganized Z lines, sarcomeres disruption areas and an increase in collagen deposition. Also, intermyofibrillar mitochondria, organized triads and myelin figures were observed (Fig. 4).

### SE intervention in pregnancy in non-diabetic rats

The diameter of RAM fibers was significantly lower in the NDex group compared with NDsed group. However, there was no significant difference in total muscle area and fiber area between both groups (Fig. 2A,B).

Immunohistochemical analysis showed that the slow-twitch fiber area and diameter were higher in NDex compared with NDsed (Fig. 2C). However, the total fast-twitch fiber area and fast-twitch fiber area and diameter were similar in NDex compared with NDsed (Fig. 2D). There was no difference in the total collagen area and type I/III collagen ratio between the groups (Fig. 3A,B). However, type I but not type III collagen area was increased in NDex compared to NDsed (Fig. 3C).

Electron micrographs of RAM from NDsed group showed disorganized Z lines, intermyofibrillar mitochondria, organized triads, and myelin figures associated with degenerated organelles (Fig. 4). However, the analysis of micrographs in samples from NDex group showed abundant intermyofibrillar mitochondria, organized triads, and myelin figures and organized Z lines.

### SE in pregnancy in diabetic rats

In diabetic exercised rats, the fiber area and diameter was higher in Dex compared with Dsed group (Fig. 2A,B). However, the total muscle area was similar in both groups.

The immunohistochemical analysis showed that the slow-twitch fiber area and diameter (Fig. 2C) and the fast-twitch fiber area and diameter (Fig. 2D) were higher in Dex group compared with Dsed group. The total collagen area and type III collagen area were unaltered in Dex compared to Dsed group (Fig. 3A–C). However, type I collagen area and collagen I/III ratio in Dex were lower compared with Dsed group.

Ultrastructural analysis showed that Dex group presents with abundant intermyofibrillar mitochondria, disorganized Z lines, sarcomeres disruption areas, and increase in collagen deposition, as well as organized triads and myelin figures compared with RAM samples from Dsed group (Fig. 4).

### SE in pregnancy in Dex, NDsed, NDex and Dsed

The fiber area and diameter were higher in Dex group compared with NDex group (Fig. 2A,B). However, the total muscle area was lower in Dex compared with NDex group.

The immunohistochemical analysis showed that the slow-twitch fiber area and diameter (Fig. 2C) and fast-twitch fiber area and diameter (Fig. 2D) were higher in Dex group compared with NDsed group. The total collagen area, type I collagen and collagen I/III ratio (Fig. 3A–C) were unaltered in Dex compared with Ndsed and NDexgroups. However, type III collagen area were higher in Dex group compared with NDsed group.

These results evidence that MHP rats after SE during the 3 weeks of pregnancy in a swim tunnel for up to 60 min/day, 6 days/week, that the study group (Dex) displays similar fiber area and diameter, slow-twitch, fast-twitch fiber area and diameter, total collagen area and type I/III collagen ratio compared to NDsed group (Figs. 2 and 3).

## Discussion

We investigated whether swimming exercise (SE) would alter the RAM DiM in rats. This preclinical study of SE started on the first day in MHP rats detailed the reversal of RAM skeletal atrophic and stiff muscle through an integrative morphological, ultrastructural and extracellular matrix (ECM) assessment. The main findings of the present study are as follows: first, the RAM DiM was confirmed, demonstrated by lower total muscle area and fiber diameter and increased type I collagen and type III collagen deposition in Dsed compared with NDsed group. These findings were already reported by our group. Second, SE during the whole pregnancy in MHP rats model reversed the RAM skeletal atrophy through an increase in fiber area and diameter, slow-twitch fiber area and diameter, fast-twitch fiber area and diameter in Dex group compared with NDsed group; and reduction in type I collagen area and type I/III collagen ratio compared with Dsed. Also, the similarity in total collagen area, type I collagen area and type I/III collagen ratio with NDsed rats were demonstrated. The close resemblance between RAM morphological, and ECM profile of MHP rats model submitted to SE (Dex group) and NDsed, suggest that SE since the beginning of pregnancy reversed the DiM. These findings support the hypothesis that DiM is inhibited by 21 days of SE training program through the entire pregnancy. Indeed, SE training plays a critical role in the reversion of DiM. It may be a sufficient duration and intensity to induce recovery of morphological changes in skeletal muscle.

Women who develop GDM are more likely to develop type 2 diabetes and UI later in life^5–7,32^. The underlying mechanism linking GDM and long-term UI seems to be diabetic myopathy, characterized by loss of muscle mass and strength ^33,34^. Many clinical and experimental studies have associated both type 1 and type 2 diabetes to muscle structural changes, including a reduction in myofiber and myofibrilar diameter, reduced muscle mass ^3,33^, reduced muscle fiber size ^35^, and decreased capacity to repair from damage ^33^.

MHP rats is an experimental model that induces structural changes including reduced muscle area and increased slow-twitch fiber in pregnant rats in RAM ^10^ and in urethral striated muscle ^8,9^, either in women or in rats ^11^. These structural harms may be related to functional changes as well as a decline in muscle strength and increased fatigability, which contributes to decreased physical capacity ^3,35^. Therefore, this damage to muscles involved in urinary continence may contribute to increased incidence of PS-UI, a predictor of long-term UI in previous GDM women ^5^. These findings allow us to adopt MHP’ rats model for studying the effects of SE on the RAM fiber-ECM structure, as it mimics GDM in women ^11^. Therefore, further study is necessary to elucidate the influence of exercise types and duration on the muscle fiber cross-sectional area of MHP rats' skeletal muscle.

SE started at the first day of pregnancy in MHP rats may be considered a preclinical study being developed as a prevention strategy of hyperglycemia in pregnancy for long-term UI. Our intention was for longer-lasting interventions leading to significant downstream impact in promoting long-term women’s health. The goals of this early intervention program in pregnancy are related to improving future maternal health in GDM women, one of the United Nation’s Eight Millennium Development Goals (MDGs) ^36,37^.

Pregnancy is the center of a program established by the International Federation of Gynecology and Obstetrics (FIGO) in the Non-Communicable Disease (NCD)—as early as possible but focusing on the first-trimester and follow-up post-pregnancy for mother and offspring, leading to prevent NCD. Pregnancy, in particular the first trimester, receive not only substantial attention but also many points of intervention, from pre-conception to postpartum ^37^. However, there are few studies evaluating the impact of hyperglycemia in pregnancy on structural muscles damage involved in urinary continence. This study tested the hypothesis that SE daily, since the beginning of pregnancy in MHP rats could act as a protective non-pharmacological intervention, so we studied its effects on RAM muscle looking at the reversal of muscle atrophy and stiffness.

Increased muscle fiber cross-sectional area, fiber area and diameter in RAM of diabetic pregnant rats after 21 days of SE is similar to the reported after 8 weeks of aerobic training ^38^. The results of this study revealed that SE daily, since the beginning of pregnancy in MHP rats, reverses the muscle atrophy caused by the maternal hyperglycemia environment. In short, these findings indicated that SE could reverse muscle atrophy in MHP rats, back into the NDsed group profile. In contrast, sarcomeres disruption areas were observed in both diabetic groups, which means that exercise had no impact on this parameter. Previous results demonstrate that RAM exposed to a hyperglycemic environment is characterized by a decrease in the number and area of the fast fiber and an increase in the number of slow fibers ^10^. In our study, daily SE during all pregnancy had an effect not only on increasing fast-twitch fiber area and diameter but also on slow-twitch fiber area and diameter of RAM muscle in both exercised groups.

The present study shows that both exercised groups regardless of diabetes had increased fast-twitch fiber area and diameter. These findings support the possibility that DiM model is inhibited by exercise training ^39^. Different SE modalities increased diameter of both slow-twitch and fast-twitch fibers on gastrocnemius and soleus muscles in the type 2 diabetes animal model ^28^. Thus, the high slow-twitch fiber area and diameter in the RA muscle of both exercised groups regardless of diabetes and high fast-twitch fiber area and diameter in diabetic exercised rats could be attributed to physiological adaptations from aerobic exercise during the whole pregnancy. Consistent with previous studies, we demonstrated that SE training during the whole pregnancy induces an increase not only in the cross-sectional area and slow-twitch fiber, but also the fast-twitch fiber ^40,41^. Indeed, SE applied in this study showed a significant reversal of muscle atrophy in pregnant hyperglycemic rats.

Exercise training plays an important role in the mitigation of muscular atrophy in DiM. Coherent with previous studies, a resistance training protocol enhanced muscle strength on extensor digitorum longus muscle, gastrocnemius and soleus muscles of male diabetic rats, improving muscle physical and motor function, minimizing the deleterious effect of diabetes on muscles^29^. Conversely, the fast and slow-twitch fiber area and diameter increase may be related to improved muscle endurance, possibly caused by mitochondrial biogenesis and increased mitochondrial enzymes activities. These findings enhance aerobic capacity and provide muscle contraction with less energy expenditure and greater oxygen uptake ^42^.

The urethral extracellular matrix also plays a role in the mechanism of urinary continence ^43^. In prior translational studies in rats, it was shown that diabetes during pregnancy results in damaged extracellular matrix (ECM) and urethral striated muscle suggestive of the high long-term UI prevalence in women ^8,9^. Other studies examined the relationship between ECM components (particularly collagens) and diabetes, PFDs and pregnancy, although few comparative data are available ^18^. The results in the present study show that SE in MHP rats did not reduce the total collagen area compared with the three other groups. However, SE in MHP rats decreases type I collagen area to similar levels of non-diabetic sedentary group and maintain high levels of type III collagen similar to SE MHP and SE non-diabetic group. The decrease in collagen type I/III ratio in SE-MHP suggests the reversal of a stiff into a soft healthy skeletal muscle. Our findings complement the results of four previously experimental studies, either in RAM or PFM ^8,10,18,43^ indicating a complete muscle remodeling. It confirms the importance of this biological approach in DiM. This changeover in collagen type I/III ratio in SE in MHP favors normal soft RAM muscle, with likely normalization of biomechanical properties in muscles involved in urinary continence. All these changes allow suggesting SE for assessment as a potential treatment for maternal UI injured by the maternal hyperglycemic environment.

Although this set of results related to SE in mitigating the negative effects of the maternal hyperglycemic environment on DiM in the transformation into a soft health muscle, the molecular regulations of this gain muscle mass is unclear. It was suggested that distinct mechanisms regulate skeletal muscle mass recovery and hypertrophy ^44^. Further studies should address this fundamental mechanism. This study as a preclinical model in the obstetrical area, the maternal-placental and fetal (MPF) unit needs to be carefully considered. Although we conducted this experiment by using the same swimming protocol established previously ^45–47^, the whole MPF unit analysis was not performed. Thus, our successful results with SE in MHP rats environment may be analysed with caution to be considered in GDM women. Also, the present study has limitations regarding the experimental diabetes induction, which represents GDM blood glucose levels, but occurred before pregnancy ^47–49^. In addition, the guidelines recommend that pregnant women should exercise at least 150 min/week or 30 min/day most days of the week ^22,23,25^. Also, specific analysis of aerobic capacity and muscle function should be explored.

In conclusion, hyperglycemia exerts an impact on DiM generating an atrophic and stiff muscle. SE intervention training during whole pregnancy in rats imposes a positive influence on DiM being capable of reversing this muscle atrophy and transforming a stiff into a soft healthy skeletal muscle. Therefore, these findings of reversal myopathy suggest that SE training during the whole pregnancy may play a therapeutic role in regulating the damages caused by the maternal hyperglycemic environment and may be considered as a potential treatment for RAM damage caused by GDM.

## Materials and methods

This current project corresponds to the translational part of the Diamater Study, a cohort Thematic Project with the financial support of São Paulo Research Foundation (FAPESP 2016/01743-5), a state research foundation in Brazil. The Diamater cohort project has been developed to investigate biomolecular muscle profiles as predictors for long-term urinary incontinence in women with Gestational Diabetes Mellitus ^4^. According to the requirement to develop innovative treatments for DiM, this translational project was developed.

### Ethics and animals

All animal experiments were approved by the Institutional Animal Care and Use Committee, Faculdade de Filosofia e Ciências, São Paulo State University (UNESP), in accordance with the Brazilian Council for Control of Animal Experimentation (CONCEA) (protocol number 007/2016). The study is reported in accordance with the ARRIVE guidelines.

**Figure 5:**
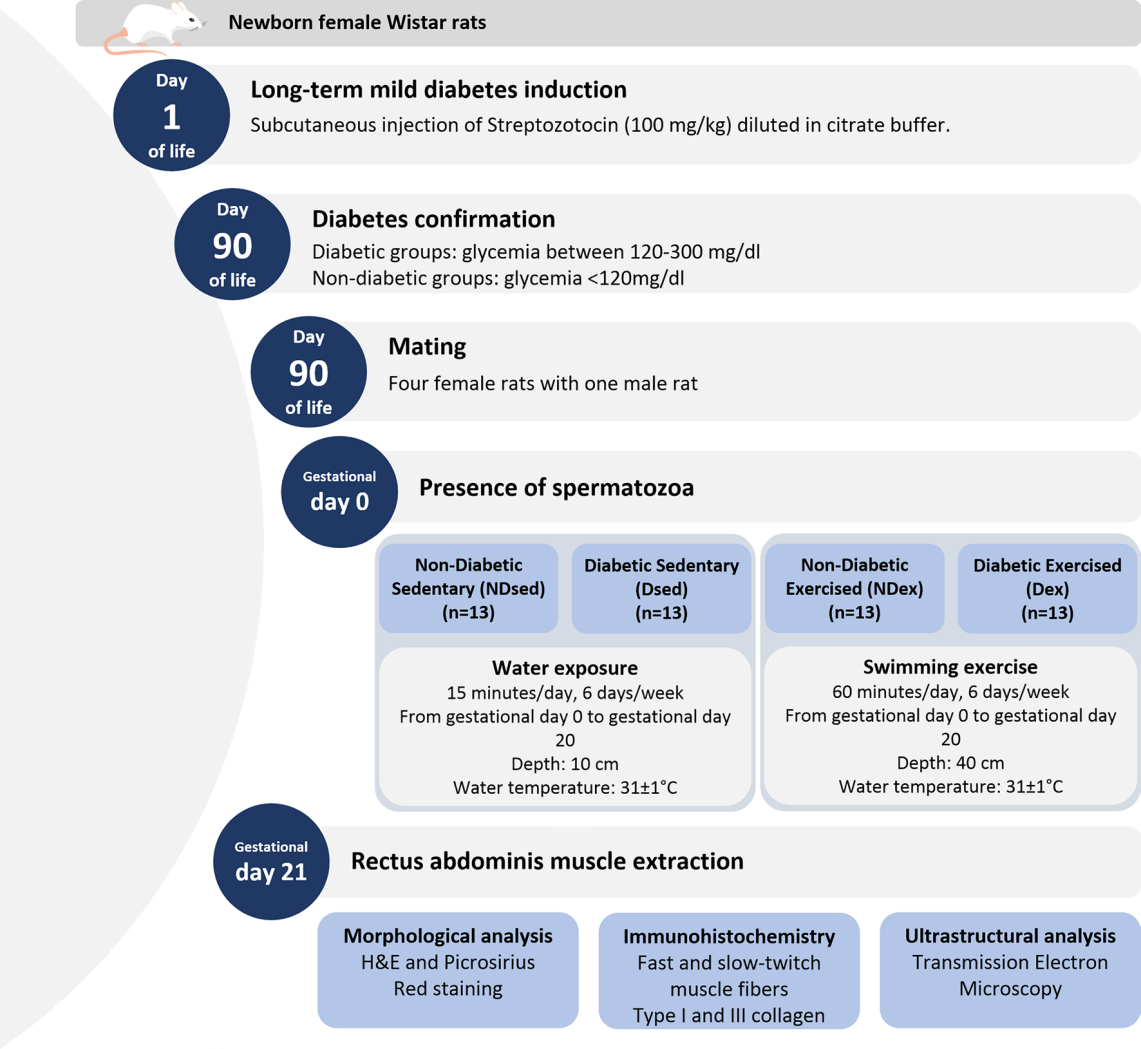
Experimental sequence of groups.

Female and male Wistar rats obtained from ANILAB were housed in a facility with constant temperature (22 ± 2 ˚C) and humidity (55 ± 5%) on a controlled 12 h light–12 h dark cycle with food and water ad libitum, in individual plastic cages during all experimental protocol. The experimental sequence is shown in Fig. 5.

### Generation of MHP rats model

The MHP model is the same as previous reports ^9–11,46,47^**.** To induce MHP rats’ model, newborn female Wistar received in the first day of life subcutaneously injection of STZ (Sigma^®^) diluted in citrate buffer (0.1 mol/l pH 4.5) in a dose of 100 mg/kg. Non-diabetic rats received subcutaneous injection of citrate buffer (0.1 mol/l pH 4.5) ^50^. All female newborn rats were maintained with their mothers until the end of the lactation period (21 days). After this period, the mother rats were euthanized by sodium thiopental injection(Thiopentax^®^—80 mg/kg). The female newborns were maintained until adulthood. Fasting blood glucose level was determined in adult life, and used for inclusion or exclusion criteria in the study. Diabetic animals should present blood glucose level between 120 mg/dl and 300 mg/dl, and non-diabetic animals blood glucose level < 120 mg/dl ^51^.

### Mating process

At approximately day 90 of age, four diabetic and non-diabetic female rats were housed overnight with one adult male rat. The presence of spermatozoa in the vaginal smear was considered gestational day 0 [52]. After, rats were housed in individual cages until 21º day of pregnancy.

### Experimental groups

On gestational day 0, female rats were randomly allocated into four experimental groups according to sedentary lifestyle or swimming exercise: Non-Diabetic Sedentary (NDsed) (n = 13), Non-Diabetic Exercised (NDex) (n = 13), Diabetic Sedentary (Dsed) (n = 13) and Diabetic Exercised (Dex) (n = 13). Dex is the study group and the other three (NDsed, NDex and Dsed) are control groups.

### Swimming exercise protocol

The swimming exercise protocol was based on previous studies ^45–47^ and considered to be a moderate intensity exercise protocol ^45^. The exercised animals were exposed to water daily temperature 31 ± 1°C for 6 days/week, from gestational day 0 until gestational day 20 on a pool at a depth of 40 cm at the water. The first training session started with 20 min, progressively increasing 10 min/day until 60 min. The sedentary rats were exposed to water daily for 15 min, at a depth of 10 cm at water temperature 31 ± 1 °C for 6 days/week, from gestational day 0 until gestational day 20, aiming not to promote physiological adaptations from exercise practice.

### Oral glucose tolerance test (OGTT)

On gestational day 17, an OGTT was performed to confirm the glucose intolerance in diabetics ^50^. Fasting glycemia and at 10, 20, 30, 60 and 120 min after administration of an intragastric glucose solution (0.2 g/mL) in a dose of 2.0 g/kg were measured. Diabetes diagnosis was confirmed with two or more blood glucose measurements > 140 mg/dL ^51^.

### RAM tissue extraction

At the end of pregnancy (gestational day 21) the dams were euthanized by sodium thiopental injection (Thiopentax, Brazil, 80 mg/kg dose). An abdominal incision was performed for RAM sample collection. The lower third on the right side of RAM was exposed, dissected, and removed for integrative morphological analysis. The fragments had approximately 0.25 cm^2^. Then, a C-section was performed and fetuses and placentas were separately analyzed in different projects.

### Integrative morphological analysis

The samples obtained were selected into separate parts according to methodological procedures. The integrative morphological analysis is composed by morphological, morphometric, immunohistochemistry and ultrastructural RAM analysis.

#### Morphological analysis

For morphological and morphometric analysis, RAM samples were immersed for 24 h in neutral 10% buffered formaldehyde, transferred to 70% alcohol and maintained at room temperature and then embedded in paraffin. The 4-µm-thick sections were cut in microtome (Reichert-Jung model 820) and fixed on microscope glass slides stained with Hematoxylin & Eosin (H&E) and Picrosirius Red. H&E-stained slides were used to observe the general morphology of the RAM. Picrosirius Red-stained slides were analyzed with the color-segmentation method to determine the red (collagen) and yellow (muscle fiber) -stained tissue in the same section and used to determine muscle, fiber and collagen area and fiber diameter. The slides were analyzed in a light microscope (Olympus Corporation^®^/BX41TF coupled with DP25-4 digital câmera). The photographs were obtained with *cell Sens* Ver 1.18 Olympus Corporation^®^ software.

#### Morphometric analysis

For morphometric analysis of muscle and collagen area, 40 sections/group (5 animals/group, 8 sections/animal) were selected. For morphometric analysis of fiber area and diameter, 100 muscle fibers/group were selected. All analyzes were performed using CellSens Dimension Version 1.16 (Olympus Corporation^®^) image analysis software (20 × magnification).

#### Immunohistochemistry

Immunohistochemical analysis was used to stain slow-twitch and fast-twitch muscle fibers and type I and III collagen. For immunohistochemistry (N = 5 samples/group), the samples were immersed for 24 h in neutral 10% buffered formaldehyde, transferred to 70% alcohol and maintained at room temperature and then embedded in paraffin. The 4-µm-thick sections were cut in the microtome. Sections were deparaffinized and incubated with antigenic recovery for 35 min in a pressure cooker. Endogenous peroxidase was blocked using H_2_O_2_ in phosphate buffered saline (PBS). After washing, the sections were incubated with bovine serum albumin solution (BSA 3%) for 1 h. The sections were incubated overnight at 4 °C with primary antibodies against myosin heavy chain, slow (Novocastra, NCL-MHCs, #6009289, 1:20) and fast (Novocastra, NCL-MHCf, #6006929, 1:20) muscle fibers, type I Collagen (Sigma Aldrich, SAB4200678, #107M4839V, 1:100) and type III Collagen (Novus Biologicals, COL3A1, #G0809, 1:100). After incubation, the sections were washed three times for five minutes with PBS and incubated with secondary antibodies for 1 h 30 min (Goat Anti-Mouse, Abcam, 1:200). For staining, the sections were incubated with diaminobenzidine for 1 min and hematoxylin for 10 min. The slow-twitch and fast-twitch muscle fibers, and type I and III Collagen were analyzed using a light microscope (Olympus Corporation^®^/BX41TF coupled with DP25-4 digital câmera) and 40 sections/group (5 animals/group, 8 sections/animal) were selected to quantify the fiber type area and collagen type area. Also, each muscle fiber was manually selected (~ 200 slow fibers/group; ~ 330 fast fibers/group) and fiber type area and diameter were quantified using CellSens Dimension (Olympus Corporation^®^) Version 1.16 image analysis software (20 × magnification). The fiber type area was quantitatively determined, as described previously^10^.

#### Ultrastructural analysis

As previously described*,* the RAM tissues obtained to ultrastructural analysis (3 samples/group) were cut into small strips and immediately immersed in Karnovsky fixative for 3 h at room temperature and transferred to the refrigerator to post-fixation in 1% osmium tetroxide for 24 h and afterward, the samples were embedded in epoxy resin. Ultra-thin sections were obtained at a longitudinal orientation and stained sections were examined using transmission electron microscopy using (JEM 1400, JEOL®).

### Statistical analysis

GraphPad Prism® v.8.0 software was used to analyze the data. Data are expressed as mean ± standard deviation (SD). Comparisons of measurements among groups were performed by two-way ANOVA followed by Tukey’s multiple comparison tests. For all statistical comparisons, p < 0.05 was considered statistically significant.

## Data Availability

The datasets used and/or analyzed during the present study are available from the corresponding author on request.
